# Kidney absorbed radiation doses for [^177^Lu]Lu-PSMA-617 and [^177^Lu]Lu-PSMA-I&T determined by 3D clinical dosimetry

**DOI:** 10.1097/MNM.0000000000001658

**Published:** 2023-01-04

**Authors:** Maike J.M. Uijen, Bastiaan M. Privé, Carla M.L. van Herpen, Harm Westdorp, Willemijn A. van Gemert, Maarten de Bakker, Martin Gotthardt, Mark W. Konijnenberg, Steffie M.B. Peters, James Nagarajah

**Affiliations:** a Department of Medical Oncology; b Department of Medical Imaging, Radboud University Medical Center, Radboud Institute for Molecular Life Sciences, Nuclear Medicine, Nijmegen; c Department of Radiation Oncology; d Department of Radiology and Nuclear Medicine, Erasmus Medical Center, Rotterdam, The Netherlands

**Keywords:** dosimetry, [^177^Lu]Lu-PSMA, prostate-specific membrane antigen (PSMA), radioligand therapy, SPECT imaging

## Abstract

For prostate-specific membrane antigen-directed radioligand therapy (PSMA-RLT), [^177^Lu]Lu-PSMA-617 and [^177^Lu]Lu-PSMA-I&T are the currently preferred compounds. Recent preclinical studies suggested ~30x higher kidney absorbed dose for [^177^Lu]Lu-PSMA-I&T compared to [^177^Lu]Lu-PSMA-617, which may lead to an increased risk of kidney toxicity. We performed two single-centre, prospective dosimetry studies with either [^177^Lu]Lu-PSMA-617 or [^177^Lu]Lu-PSMA-I&T, using an identical dosimetry protocol. We evaluated the absorbed doses of both ^177^Lu-labelled radioligands in human kidneys.

**Methods:**

3D SPECT/computed tomography (CT) imaging of the kidneys was performed after PSMA-RLT in cancer patients with PSMA-positive disease and an adequate glomerular filtration rate (≥50 mL/min). Ten metastatic hormone-sensitive prostate cancer patients (mHSPC) were treated with [^177^Lu]Lu-PSMA-617 and 10 advanced salivary gland cancer (SGC) patients were treated with [^177^Lu]Lu-PSMA-I&T. SPECT/CT imaging was performed at five timepoints (1 h, 24 h, 48 h, 72 h, and 168 h post-injection). In mHSPC patients, SPECT/CT imaging was performed after cycles 1 and 2 (cumulative activity: 9 GBq) and in SGC patients only after cycle 1 (activity: 7.4 GBq). Kidney absorbed dose was calculated using organ-based dosimetry.

**Results:**

The median kidney absorbed dose was 0.49 Gy/GBq (range: 0.34–0.66) and 0.73 Gy/GBq (range: 0.42–1.31) for [^177^Lu]Lu-PSMA-617 and [^177^Lu]Lu-PSMA-I&T, respectively (independent samples *t* test; *P* = 0.010).

**Conclusion:**

This study shows that the kidney absorbed dose for [^177^Lu]Lu-PSMA-617 and [^177^Lu]Lu-PSMA-I&T differs, with a ~1.5x higher median kidney absorbed dose for [^177^Lu]Lu-PSMA-I&T. This difference in the clinical setting is considerably smaller than observed in preclinical studies and may not hamper treatments with [^177^Lu]Lu-PSMA-I&T.

## Introduction

Prostate-specific membrane antigen (PSMA) is a transmembrane protein and highly overexpressed by prostate cancer cells, which makes it an ideal target for theranostic application. PSMA-radioligand therapy (PSMA-RLT) with [^177^Lu]Lu-PSMA-617 and [^177^Lu]Lu-PSMA-I&T showed promising response rates in metastatic castration-resistant prostate cancer (mCRPC) patients, with a favourable toxicity profile [1,2]. Following these outcomes, PSMA-RLT is also studied for other PSMA-expressing cancers, such as salivary gland cancer (SGC) [3,4].

Although [^177^Lu]Lu-PSMA-617 and [^177^Lu]Lu-PSMA-I&T have the identical PSMA binding motif (glutamate–urea–lysine), they differ with respect to the linker and chelator resulting in different chemical properties [5]. In humans, [^177^Lu]Lu-PSMA-617 and [^177^Lu]Lu-PSMA-I&T have not been compared head-to-head.

Unfortunately, the intestines, salivary glands, and proximal tubule of the kidneys also show high uptake of PSMA ligands, possibly resulting in significant radiation doses to these healthy organs following PSMA-RLT. Moreover, [^177^Lu]Lu-PSMA-617 and [^177^Lu]Lu-PSMA-I&T are renally excreted, which may increase the radiation exposure to the kidneys even further. The European Guidelines also identified the kidneys as the most important dose-limiting organ for PSMA-RLT [6].

While kidney failure due to PSMA-RLT is rarely seen, this might also be the result of the poor overall survival of the end-stage patients that currently received PSMA-RLT. However, the number of trials that investigate PSMA-RLT in early-stage cancer patients is increasing (e.g. NCT04720157, NCT04430192, and NCT04443062) [7,8]. In these patients, late toxicities may become apparent during longer follow-ups, such as kidney-related toxicities. Moreover, doses to the healthy organs such as the kidneys are important as organ toxicities could reduce the quality of life of patients and preclude patients from qualifying for the following treatment lines.

Preclinical studies showed that kidney radiation doses with [^177^Lu]Lu-PSMA-I&T are approximately 30 times higher compared to [^177^Lu]Lu-PSMA-617 [9,10], absorbed dose in mice resulted in ~8.5 Gy with 30 MBq [177Lu]Lu-PSMA-I&T versus ~0.25 Gy with 30 MBq [177Lu]Lu-PSMA-617 [9]. This suggests an increased risk of kidney toxicity with [^177^Lu]Lu-PSMA-I&T. However, these preclinical experiments were performed using *in vitro* and in murine models which do not directly translate to human kidneys.

Furthermore, in contrast to these preclinical findings, several clinical dosimetry studies found a comparable mean kidney-absorbed radiation dose for ^177^Lu-PSMA-617 and ^177^Lu-PSMA-I&T [11–18]. Unfortunately, these studies applied varying dosimetry protocols, often only using planar scans, and are therefore difficult to compare. Thus, it is presently unclear if patients receiving [^177^Lu]Lu-PSMA-I&T are exposed to higher kidney radiation doses compared to [^177^Lu]Lu-PSMA-617. In this study, we compared the kidney dosimetry results of [^177^Lu]Lu-PSMA-I&T and [^177^Lu]Lu-PSMA-617 which were acquired from two prospective clinical trials, following an identical 3D dosimetry protocol.

## Material and methods

### Patients

In a third-line academic institute (Radboudumc, the Netherlands), two prospective clinical studies were conducted on PSMA-RLT in cancer patients with PSMA-positive disease and an adequate glomerular filtration rate (GFR) (≥50 mL/min). Both studies used an identical dosimetry protocol. One study applied a first cycle of 3 GBq and a second cycle (after 6 weeks) of ~6 GBq [^177^Lu]Lu-PSMA-617 in ten low-volume metastatic hormone-sensitive prostate cancer (mHSPC) patients, thus in total a cumulative activity of ~9 GBq [7]. The other used ~7.4 GBq [^177^Lu]Lu-PSMA-I&T in 10 advanced SGC patients (NCT04291300). The dosimetry protocol of both trials consisted of five time points (1 h, 24 h, 48 h, 72 h, and 168 h) 3D SPECT/CT imaging post [^177^Lu]Lu-PSMA injection. All scans were acquired on a Symbia T16 or Symbia Intevo Bold system (Siemens Healthineers, Erlangen, Germany) using a medium-energy low-penetration collimator, a 20% photon energy window at 208 keV with dual-energy window for Compton scattering, 64 projections per detector and 14 s per projection, matrix size 128 × 128 and zoom 1. Data were reconstructed using ordered subsets maximization expectation reconstruction (Flash 3D with collimator detector response) using four iterations, eight subsets and a smoothing Gaussian filter of 8.4 mm.

### Dosimetry analysis

The absorbed doses for both cohorts were calculated in a similar way, as previously described [19]. In short, volumetric organ-based dosimetry was performed according to the scheme defined by the Committee on Medical Internal Radiation Dose [20] using Hermes HybridViever/Dosimetry (Hermes Medical Solutions, Stockholm, Sweden). All SPECT/CT images were co-registered per patient, followed by drawing volumes of interest of the kidneys. Kidney absorbed radiation dose was determined in Olinda 2.1 (Hermes Medical Solutions, Stockholm, Sweden) using gender-specific human kidney weights based on the ICRP Publication 89 [21], corresponding S-values and a mono-exponential fit.

### Statistical analysis

To test for baseline differences between study populations, the independent samples *t* test was used for continuous variables and Fisher’s exact test was used for categorical variables. The independent samples *t* test was used to compare the kidney absorbed radiation dose between [^177^Lu]Lu-PSMA-617 treated mHSPC patients and [^177^Lu]Lu-PSMA-I&T treated SGC patients. A *P* value <0.05 was considered statistically significant. Statistical analyses were performed using IBM SPSS Statistics version 25.0 (IBM Corp, Armonk, New York, USA).

## Results

A summary of both clinical studies is provided in Table 1.

**Table 1:** ^177^Lu-PSMA treatment and dosimetry imaging

	mHSPC (*n* = 10)	SGC (*n* = 10)
PSMA ligand for PSMA-RLT	PSMA-617	PSMA-I&T
^177^Lu-PSMA-RLT treatment	cycle 1: 3 GBq	2–4 cycles
	cycle 2: 6 GBq	of 7.4 GBq
Dosimetry imaging	After	After
	cycle 1 + cycle 2	Cycle 1
Cumulative activity^a^	9 GBq	7.4 GBq
Dosimetry imaging timepoints (post-injection)^b^	1 h	1 h
	24 h	24 h
	48 h	48 h
	72 h	72 h
	168 h	168 h
Clinical study	NCT03828838	NCT04291300

mHSPC, low-volume metastatic hormone-sensitive prostate cancer patients; SGC, salivary gland cancer patients; PSMA, prostate-specific membrane antigen; RLT, radioligand therapy; ^177^Lu, lutetium-177; GBq, Giga-becquerel.

a Total amount of activity for which dosimetry imaging data is available.

b This included SPECT/CT imaging of the kidneys.

### Patient characteristics

**Fig. 1:**
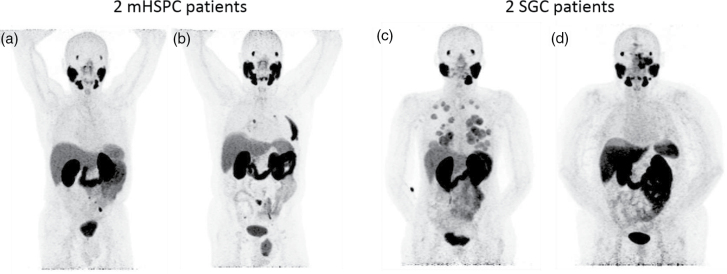
[^68^Ga]Ga-PSMA-11 PET maximum intensity projections (MIP) before PSMA-RLT treatment. (a) mHSPC patient with oligo-recurrent disease following surgery and external beam radiotherapy. (b) mHSPC patient with nine lymph node and bone metastases following radical external beam radiotherapy. Note the inguinal herniation with uptake of [^68^Ga]Ga-PSMA-11. (c) SGC patient primary tumour arose from right submandibular gland (status post-surgery), with lung and liver metastases. (d) SGC patient primary tumour arose from left lacrimal gland, with an incurable local tumour and lymph node metastasis (near left submandibular gland). mHSPC, metastatic hormone-sensitive prostate cancer; SGC, salivary gland cancer.

Per protocol, all 20 patients had adequate kidney function at baseline (see Table 2). The kidney uptake on baseline ^68^Ga-PSMA-11 PET was also comparable between the two populations. The SGC patients had a significantly higher tumour burden than the low-volume mHSPC patients (*P* ≤ 0.001). Figure 1 illustrates the baseline disease burden of four patients (two mHSPC and two SGC). Furthermore, other baseline patient characteristics are presented in Table 2.

**Table 2:** Baseline patientcharacteristics

	mHSPC (*n* = 10)	SGC (*n* = 10)	*P* value
	No. patients (%)	No. patients (%)	
Gender			**0.033**
Male	10 (100)	5 (50)	
Female	0 (0)	5 (50)	
Age, median (range)	67 (61–77)	64 (51–74)	0.192
Disease burden			**<0.001**
≤10 tumour lesions	10 (100)	1 (10)	
>10 tumour lesions	0 (0)	9 (90)	
Kidney function^a^			**0.006**
eGFR^b^ (mL/min), median (range)	71 (61–88)	90 (61–90)	
Kidney uptake ^68^Ga-PSMA-11 PET^a^^,^^c^			
SUVmax, median (range)	60.5 (35.7–97.4)	59.4 (23.5–72.9)	0.312
SUVmean, median (range)	32.2 (16.9–51.2)	31.0 (12.1–40.0)	0.602
Median kidney VOI volume (mL) on SPECT/CT (range)	190 (130–250)	198 (160–295)	0.408

Bold values are statistically significant (*P* < 0.05).

eGFR, estimated glomerular filtration rate; ^68^Ga, Gallium-68; PSMA, prostate-specific membrane antigen; mHSPC, low-volume metastatic hormone-sensitive prostate cancer patients; SGC, salivary gland cancer patients; SUVmax, maximum standardized uptake value; SUVmean, mean standardized uptake value; VOI, volume of interest.

a Maximum time-interval between baseline kidney function assessment and baseline ^68^Ga-PSMA-11 PET with the start of ^177^Lu-PSMA RLT was 4 weeks.

b eGFR: based on the CKD-EPI equation.

c Time interval between ^68^Ga-PSMA injection and imaging was ±1 h. ^68^Ga-PSMA dose was 2.0 MBq/kg ± 10%, with a minimum of 20 Mbq and a maximum of 300 Mbq.

### Kidney-absorbed radiation doses

**Fig. 2:**
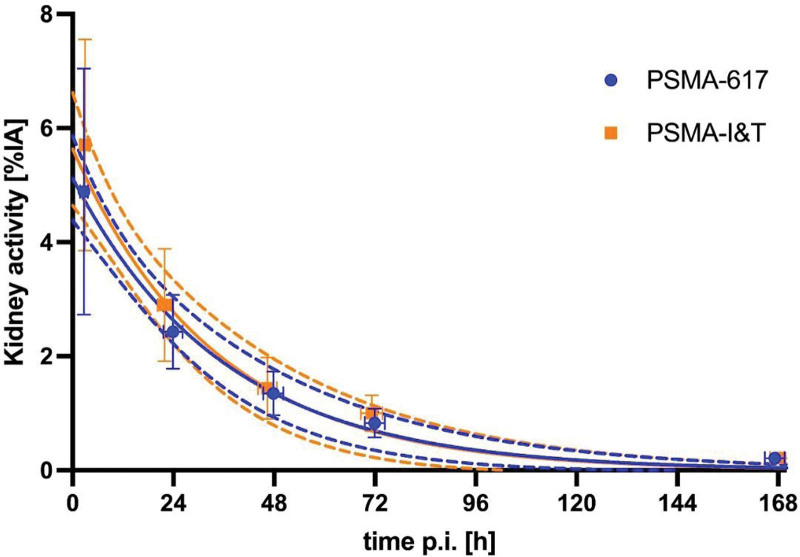
Kidney time-activity curves of [^177^Lu]Lu-PSMA-617 and [^177^Lu]Lu-PSMA-I&T. The solid lines indicate single-exponential curve fits with their 95% confidence limits shown as dashed lines. %IA, percentage of injected activity; p.i., post-injection; h, hours.

Median kidney absorbed dose was 0.49 Gy/GBq (range: 0.34–0.66) for treatment with [^177^Lu]Lu-PSMA-617, whereas the median kidney absorbed dose was 0.73 Gy/GBq (range: 0.42-1.31) for [^177^Lu]Lu-PSMA-I&T (Table 3). The difference in absorbed dose between [^177^Lu]Lu-PSMA-617 and [^177^Lu]Lu-PSMA-I&T was statistically significant (*P* = 0.010). As depicted in Fig. 2, apart from the initial higher kidney activity at the earliest timepoints with [^177^Lu]Lu-PSMA-I&T, both [^177^Lu]Lu-PSMA-617 and [^177^Lu]Lu-PSMA-I&T show comparable kinetics over time. The median clearance half-lives were 26 h (range: 15–43 h) and 20 h (range: 17–38 h), for PSMA-617 and PSMA I&T, respectively (*P* = 0.27).

**Table 3:** Kidney absorbed doses per injected activity of [^177^Lu]Lu-PSMA-617 and [^177^Lu]Lu-PSMA-I&T

Kidney absorbed dose (Gy/GBq)	mHSPC (*n* = 10) [^177^Lu]Lu-PSMA-617	SGC (*n* = 10) [^177^Lu]Lu-PSMA-I&T
Median	0.49	0.73
Range	0.34–0.66	0.42–1.31

mHSPC, low-volume metastatic hormone-sensitive prostate cancer patients; PSMA, prostate-specific membrane antigen; SGC, salivary gland cancer patients.

## Discussion

We performed two state-of-the-art 3D SPECT/CT dosimetry studies of [^177^Lu]Lu-PSMA-617 and [^177^Lu]Lu-PSMA-I&T in a prospective setting. Therefore, we were able to compare the absorbed doses by the kidneys of each respective compound most accurately to date. We observed a ~1.5x higher median kidney absorbed dose for [^177^Lu]Lu-PSMA-I&T (0.73 Gy/GBq) compared to [^177^Lu]Lu-PSMA-617 (0.49 Gy/GBq). This difference was statistically significant (*P* = 0.010). In a clinical setting, the absorbed dose to the kidneys would be 5.4 Gy (range: 3.1–9.7 Gy) vs. 3.6 Gy (range: 2.5–4.9 Gy) for 7.4 GBq [^177^Lu]Lu-PSMA-I&T or [^177^Lu]Lu-PSMA-617, respectively.

Previous preclinical studies have suggested that [^177^Lu]Lu-PSMA-I&T resulted in a much higher (30x) kidney radiation dose compared to [^177^Lu]Lu-PSMA-617 [9,22]. This was recently supported by retrospective work from Schuchardt *et al*. showing a significant difference in kidney absorbed dose between these two compounds (0.77 Gy/GBq for [^177^Lu]Lu-PSMA-617 vs. 0.92 Gy/GBq for ^177^Lu-PSMA-I&T, *P* = 0.0015) [16]. However, this retrospective study is impaired by its alternating dosimetry protocol and by relying on planar imaging, which can significantly affect the accuracy of the dosimetry outcomes [23–25]. With our results using an elaborate and identical dosimetry protocol, we can confirm the previous preclinical and retrospective study outcomes. However, the observed differences in kidney radiation doses are considerably lower than the preclinical work suggested and more in line with the retrospective study of Schuchardt *et al*. Therefore, the risk for kidney toxicity with [^177^Lu]Lu-PSMA-I&T may be of less concern in a real-life setting.

To date, the longest follow-up has been reported for [^177^Lu]Lu-PSMA-617 with a median of 30.4 months. At this time, the authors did not observe a grade *>*3 of kidney toxicity [26]. Neither did the recently published pivotal ‘VISION’ trial of [^177^Lu]Lu-PSMA-617 (median follow-up 20.9 months) [2]. However, the median follow-up in both these studies of end-stage mCRPC patients was rather short due to the poor survival in most of the patients. In addition, there is no mature data on adverse events following [^177^Lu]Lu-PSMA-I&T yet as the results of the pivotal trial of [^177^Lu]Lu-PSMA-I&T are awaited (NCT04647526) [1,27]. Therefore, the clinical consequences of a higher radiation dose for [^177^Lu]Lu-PSMA-I&T in the kidneys are to be determined.

The European guidelines suggest that the threshold dose of [^177^Lu]Lu-PSMA is 40 Gy in Biological Effective Dose (BED) before kidney-related toxicity occurs [6]. This threshold dose is mostly based on ^177^Lu-DOTATATE studies and on data from external beam radiotherapy studies. We, therefore, urge the need to include dosimetry in trials to adequately correlate adverse events to absorbed doses to the organs at risk. This will also pave the way for the broad adoption of targeted radionuclide therapies particularly in earlier-stage cancer patients and for more than a fixed amount of (4–6) cycles. After all, the dosimetry of radionuclide therapies allows for personalized dosing schemes [28].

Although it is yet unknown why the kidney uptake differs between [^177^Lu]Lu-PSMA-I&T and [^177^Lu]Lu-PSMA-617, it is postulated that this is related to the negatively charged chelator DOTAGA (-1) of [^177^Lu]Lu-PSMA-I&T compared to the neutrally charged DOTA (0) of [^177^Lu]Lu-PSMA-617. Hence, negatively charged chelators can result in higher reabsorption by the proximal tubule of the kidneys [29]. However, the degree of renal doses is also related to the structure, size, binding and circulation time of the radioligand complex [29]. Therefore, more studies are needed to elucidate the exact cause of the higher kidney doses of [^177^Lu]Lu-PSMA-I&T compared to [^177^Lu]Lu-PSMA-617. Moreover, murine tumour models have different expressions of the FOLH1 receptor in healthy tissues (such as the kidneys) compared to humans [5]. This may also skew the comparison of kidney dose in mice to humans and explain the large difference between the preclinical and clinical dosimetry data.

This study was limited by its two limited-size cohorts from two distinct malignancies with one being prostate cancer and the other SGC. However, we believe that the cancer type does not affect the kidney kinetics of [^177^Lu]Lu-PSMA-I&T or [^177^Lu]Lu-PSMA-617. Furthermore, although all 20 patients had good kidney function, the baseline GFR was dissimilar in favour of the SGC group. The consequence of this difference is to be determined. But, a recent study showed that baseline kidney function was not predictive of kidney absorbed dose for PSMA-RLT [30]. As a final note, we advocate international harmonization of dosimetry protocols to improve comparability of dose estimations worldwide.

### Conclusion

This prospective five-timepoint 3D SPECT/CT dosimetry study showed that the kidney absorbed dose significantly differed between [^177^Lu]Lu-PSMA-617 and [^177^Lu]Lu-PSMA-I&T, with a ~1.5x higher median kidney absorbed dose for [^177^Lu]Lu-PSMA-I&T. Despite our limitations (e.g. different malignancies and differences in administered activity), the difference of kidney radiation doses in the clinical setting seems considerably lower than suggested by preclinical studies. Thus, the clinical relevance of the different kidney radiation doses may be of less importance. Furthermore, the effect of PSMA-RLT on kidney function needs to be assessed in proper series with long-term follow-up.

## Acknowledgements

This work was supported by the Dutch Cancer Society (KWF), the Dutch Prostate cancer foundation, and the Radboud Oncology Foundation.

Preliminary data of this article were presented at EANM 2021.

All procedures performed in studies involving human participants were in accordance with the ethical standards of the institutional and/or national research committee and with the 1964 Helsinki Declaration and its later amendments or comparable ethical standards. Both studies study were approved by the Medical Review Ethics Committee Region Arnhem-Nijmegen and were registered on ClinicalTrials.gov.

Informed consent was obtained from all induvial participants included in the study.

The datasets generated during and/or analysed during the current study are available from the corresponding author on reasonable request.

All authors contributed to the study conception and design. Material preparation, data collection and analysis were performed by M.U., B.P., M.K., S.P. and J.N. The first draft of the article was written by MU & BP and all authors commented on previous versions of the article. All authors read and approved the final article. All authors contributed to writing this article. All authors read and approved of the final article.

### Conflicts of interest

C.M.L.v.H.: Consultant fees for participation in advisory boards (not personal, but on behalf of the institute): Bayer, Bristol-Myers Squibb, Ipsen, MSD, Regeneron, and Philips Molecular Pathway Diagnostics. Research grants: Astra Zeneca, Bristol-Myers Squibb, MSD, Merck, Ipsen, Novartis, and Sanofi. J.N.: Consultation for CURIUM, IIT Novartis and ABX. For the remaining authors, there are no conflicts of interest.
